# Lane-Petter’s Pipeline: Why reliably decreasing animal research takes more than replacements

**DOI:** 10.1007/s13194-025-00715-8

**Published:** 2026-01-29

**Authors:** Nico Dario Müller

**Affiliations:** Philosophical Seminar, University of Basel, Steinengraben 5, 4051 Basel, Switzerland

**Keywords:** Animal research, Replacement, Three Rs, Material models, Innovation, Policy

## Abstract

Programs for the “three Rs” of “replace, reduce, refine” (Russell & Burch 1959) aim to make science more compatible with animal protection. This article draws attention to a major obstacle to that endeavor: While some animal models are being replaced by alternatives, new animal models are also being developed. Animal research thus resembles a moving pipeline, not a fixed set of models, as British scientist and regulator William Lane-Petter noted over half a century ago (Lane-Petter 1961). This article provides an account of “Lane-Petter’s Pipeline” (LPP) by drawing on philosophical literature on animal models and advances two claims: First, there is practically infinite room for the innovation of new animal models, so LPP can continue to move indefinitely unless policy intervenes; Second, the extent to which innovation focuses on new animal models rather than new non-animal-models plausibly depends on scientific repertoires (Ankeny & Leonelli, 2016) and the built environment of scientific work, which policy can help to shape. This highlights the potential, and the need, for strategic policies that tilt the environment of innovation towards new non-animal-models rather than new animal models.

## Introduction

Many modern states aim to both protect animals and promote scientific research. When scientists harm animals to gain relevant data, these two goals conflict. Legal systems typically resolve such conflicts by weighing the interests at stake, a procedure called “harm-benefit analysis” (Brønstad et al., 2016; Laber et al., 2016; Olsson et al., 2024). This helps them choose which goal should be sacrificed for the other. But, even if we suppose that the harms and benefits of studies can be meaningfully weighed, that they should be so weighed, and that every single weighing is done correctly, it would be preferable not to have to choose. As Brigid Brophy pointed out: “The moral thing to do about a moral dilemma is circumvent it” (1972, p. 125; see also Müller, 2024b). States who value both animals and science thus have reason to ask: how can policy make the two more compatible over time? How can harm to animals for scientific purposes be reliably decreased without jeopardizing science?

The prevailing approach today is that of the “three Rs” of *replacing* animal models with other models, *reducing* the sample size of animal studies, and *refining* handling and husbandry to decrease harm (Russell & Burch, 1959). Across the globe, and especially in Europe, dozens of institutions and programs support the innovation and proliferation of alternative methods in line with this approach (Bayne et al., 2015; Neuhaus et al., 2022a, b). Among the “three Rs,” replacement takes a particular place of pride. Russell and Burch themselves considered it the first and most important “R” (1959, Ch. 4). The European Union’s animal experimentation directive (2010/63/EU, Article 10) names “full replacement” as an eventual long-term goal of animal experimentation policy. And various scholars and activists urge that scientists and institutions should prioritize replacement more strongly over reduction and refinement (Franco et al., 2018; Baumgartl-Simons & Hohensee, 2019; Blattner, 2019; Herrmann, 2019a, b; Anderson & Smith, 2024; Bailey, 2024). There is a palpable hope that replacement holds the promise of making science significantly more animal-friendly over time.

The present article will discuss a major obstacle to fulfilling this promise, focusing on animal model use in scientific research (not regulatory testing, education, or other contexts in which animals might be used for experiments). The obstacle is that innovation happens not just in alternatives (e.g., based on cell cultures, organs-on-a-chip, or computer simulations), but also within animal research itself (e.g., by using new species, editing genes, or tracking new properties). Scientist and regulator William Lane-Petter described this dynamic in 1961 as a “pipeline” (1961, p. 133). On one end of the pipeline, animal uses exit science because they have been made obsolete by alternatives, but on the other end, novel uses of animals enter. The dynamic will thus be termed “Lane-Petter’s Pipeline” (LPP).

This article’s main contribution is to point out LPP and the limitation it puts on replacement-oriented animal research policies. If the aim of these policies is to reliably reduce goal conflicts between science and animal protection, they need to address both ends of LPP and not just one. Replacement, although it might be the most transformative of the “three Rs,” is still focused on the exit side. It is half the solution at best. The article will ask what the other half might be – what kinds of policies would be needed to address LPP’s entry side – but does not claim to have any definite answers.

Previous literature has addressed potential strategies for animal experimentation reduction or a full phase-out (Abarkan et al., 2022; Baumgartl-Simons & Hohensee, 2019; Herrmann, 2019a, b; Marshall et al., 2022; Müller, 2024a, b, c). Furthermore, the relative importance of replacement among the “three Rs” has been discussed from philosophical and empirical standpoints (Bailey, 2024; Balls, 2020; Franco et al., 2018; Herrmann et al., 2019). Literature in the philosophy of science has provided accounts of how animal models represent their targets, how they rely on databases and physical infrastructure, and how they contribute to scientific thinking beyond just providing data (see Ankeny & Leonelli, 2020; Green, 2024 for overviews). Other contributions have addressed factors that affect researcher’s selection of animal models (Dietrich et al., 2020; Kahrass et al., 2024; Lohse, 2021; Veening-Griffioen et al., 2020). But neither the dynamic of simultaneous innovation in alternatives and new animal models nor its implications for animal research policy have been discussed in this literature yet.^1^ This is the research gap addressed by the present article.

The article will proceed as follows. First, it will consider the notion of “replacement” to argue that efforts for replacement are necessary, but not sufficient, to reliably decrease overall reliance on animal models in research (Section 2). Second, the concept of LPP will be introduced by drawing on William Lane-Petter’s own writings (Section 3). Third, two claims will be advanced about the pipeline dynamic: There is practically infinite innovation potential within animal research, so LPP can continue moving indefinitely unless policy intervenes (Section 4); and policy can address some of the factors that plausibly influence which new models get innovated (Section 5). In other words, policy *needs* to address LPP if the goal is to reliably decrease harm to animals in science, and it plausibly *can* address it.

## Replacement’s contribution to decreasing reliance on animal models

Today, the policy goal of decreasing harm to animals without holding science back is mainly pursued by means of platforms and programs for the “three Rs” of “replace, reduce, refine” (Russell & Burch, 1959; see also Bayne et al., 2015; Neuhaus et al., 2022a, b). Reduction (minimizing the sample size) and refinement (decreasing harm inflicted on animals in and around experiments) are widely viewed as shorter-term approaches aiming at damage control, while replacement is considered the more fundamentally transformative approach that can render science more animal-friendly over time (Olsson et al., 2012).

What do we mean by a “replacement?” Kramer’s (2024) account of alternatives provides a helpful starting point: X is an alternative to Y if (1) X addresses the same problem as Y, under an appropriate description of that problem; (2) X has a reasonable chance of success, compared to Y, in solving the problem; (3) X is not ethically unacceptable as a solution to the problem also addressed by Y (2024, p. 92). Replacements for animal experiments, e.g., based on cell cultures, organoids, or computer simulations, plausibly meet these conditions and thus count as alternatives. Whether reduction and refinement techniques can count as alternatives despite still harming animals depends on what ethical views are presupposed in condition (3).

Kramer’s condition (1) highlights that a method can only be an alternative for another method relative to a specific research problem. Developing an alternative thus requires the articulation and selection of a research problem already addressed by the original. This contrasts with the more flexible ways in which methods and problems often interact in biology.^2^ Rheinberger (1997) has offered many examples of biological advances that were made not primarily by postulating and testing theories, but rather by tweaking “experimental systems,” i.e., research materials and ways of manipulating and interpreting them (Rheinberger, 1997; see Weber, 2018 for an overview and discussion). For example, the in vitro system that would lead to the discovery of transfer RNA had originally been developed to study cancer cells, but researchers recognized opportunities to address new questions with it, and the very notion of transfer RNA was only innovated in the process (Rheinberger, 1997, p. 7). Problem selection does not always precede method selection, but sometimes the options provided by an experimental system only enable the articulation and selection of a new research problem.

There is thus a conceptual asymmetry in how animal models and alternatives (considered as such) relate to research problems. Animal models can both inspire *and* respond to problems, while alternatives (considered as such) merely respond. As Lohse (2021, p. 44) puts it, alternatives are used to fulfil the “primary epistemic function” of providing evidence to answer given research questions, but animal models often retain the more creative “secondary epistemic function” of inspiring new research ideas. Of course, specific alternatives such as *in vitro* models can also inspire new research ideas, but in doing so, they break out of their role as alternatives. To frame animal research transitions as a matter of developing and promoting “alternatives” thus also means limiting the role of non-animal models.^3^

Efforts to innovate and proliferate replacements for animal models can thus be understood as continuously reacting to a research agenda set by animal-reliant work. Whether, and by how much, such efforts can decrease overall reliance on animal models depends on how animal research itself develops. For example, it depends on how much animal research is funded and whether new technologies become available that create opportunities for more animal research (e.g., gene modification techniques) – factors that are far beyond any replacement program’s control (see NC3Rs, 2012, p. 51). Replacement can help to mitigate the growth of animal research, but on its own, it cannot reliably decrease the overall numbers.

An objection to the above argument could be that “replacement” can be defined more broadly, without referring to a predefined research purpose. A course offered by the European Union’s Education and Training Platform for Laboratory Animal Science (ETPLAS) presents “reformulating the research question” as a replacement technique (ETPLAS, 2021, lesson 6). For example, instead of posing a question about skin sensitization in an animal model, one might be able to reformulate it in terms of adverse outcome pathways (see Leist et al., 2017), and answer it partially or fully based on a combination of *in vitro* and *in silico* methods (ETPLAS, 2021, lesson 7). However, this merely moves the focus from a predefined *research* purpose to a predefined *practical* purpose already served by an animal-reliant method, still putting non-animal-models in a reactive position.^4^

In sum, while alternatives have a role to play in decreasing reliance on animal models, the very notion of an alternative implies that they react to a research agenda set by work that relies on animal models. Meanwhile, animal models are free to both respond to research problems and inspire their identification and articulation. It is this open-ended and exploratory character of animal research that leads to the problem of Lane-Petter’s Pipeline, to be discussed in the following section.

## The problem of Lane-Petter’s Pipeline

Various pipeline metaphors have been used to describe sequential processes involved in science – the “research pipeline” (e.g., Kleinman & Mold, 2009), the “translational pipeline” (e.g., Callard et al., 2012), the “drug discovery pipeline” (e.g., Loskill et al., 2021), but also the “STEM pipeline” that leads students to a scientific career (e.g., Cannady et al., 2014), the “leaky pipeline” that fails to keep women in academia (e.g., Windsor et al., 2021), and the “researcher’s pipeline” through which scientists turn ideas into publications (e.g., Lebo, 2016). Usually, pipeline metaphors are used to capture *desirable* processes – ones that should remain well supplied, run smoothly, and yield abundant output. They typically convey that active direction is required so that inputs (e.g., basic research, early-career women scientists, research ideas) do not disperse, but continue along the intended path.

In his book on the “Provision of Laboratory Animals for Research” (1961), scientist and regulator William Lane-Petter (for more on his career, see Kirk, 2010) used a quite different pipeline metaphor to describe the simultaneous innovation of replacements and novel uses of animals:

“[…] as time goes on certain uses for laboratory animals become obsolete, because they are replaced by other methods, and so the demand for laboratory animals is reduced. However, as uses for laboratory animals are, so to speak, drawn off in this way, new uses are being evolved. We can conceive of a pipeline, comprehending all the current uses of laboratory animals, discharging into simpler systems at one end and at the other being recharged with new developments arising out of new biological research.” (Lane-Petter, 1961, p. 133)

In contrast to other pipeline metaphors, Lane-Petter’s is not built around the idea of efficient and focused production of a specific desired output. Its emphasis rather lies on the open-endedness of the continuous conversion of animal uses into replacements – there is always more where that (i.e., animal experiments) came from. The pipeline thus implicitly contrasts with a heap or a fixed set of originals – something that could be removed bit by bit without being replenished.

**Fig. 1:**
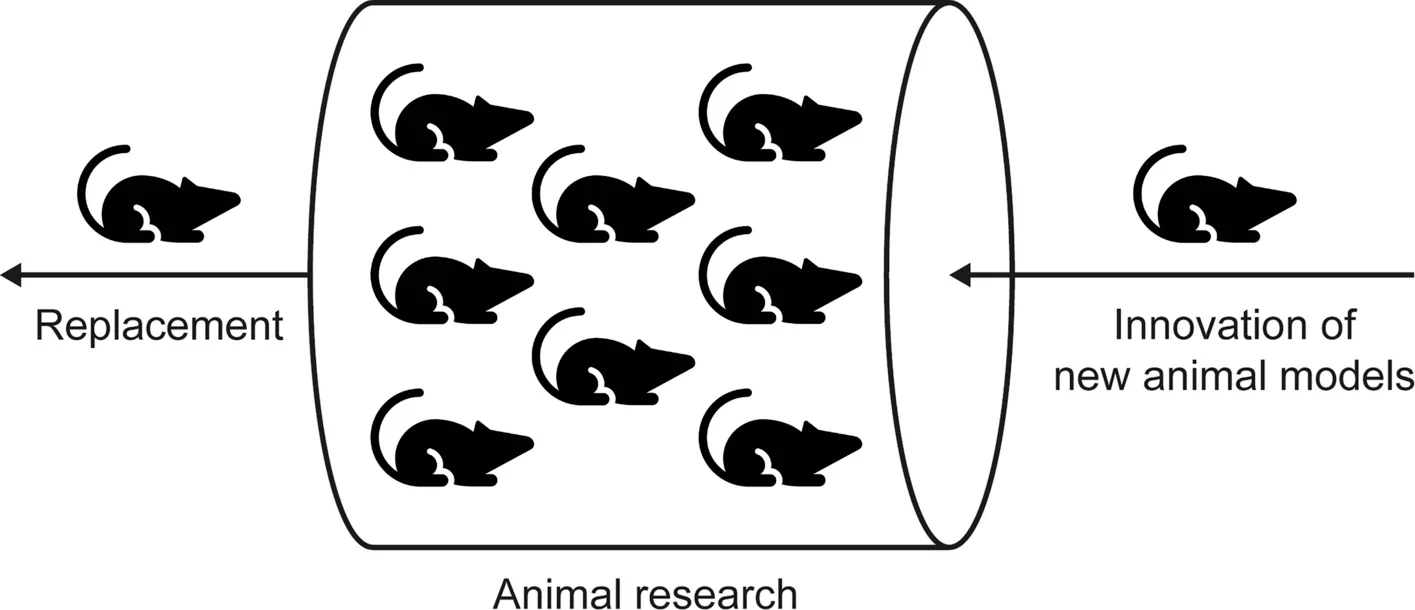
Lane-Petter’s Pipeline (LPP): Animals are leaving the pipeline of research through replacements (left), but meanwhile new animals are entering through novel animal-reliant research procedures (right)

Lane-Petter’s discussion of LPP, presented only as a passing remark, is limited in two respects. First, he discusses it exclusively as a pipeline of *animal uses*, thus, individual experimental procedures. By contrast, it may sometimes be helpful to take a more coarse-grained perspective and look at LPP as a pipeline of *animal models* (types or tokens of the animals used). Second, Lane-Petter conceives of the influx of new animal uses purely as a matter of technical innovation within animal experimentation, not as a matter of new research problems being raised. But on both counts, Lane-Petter’s concept can be extended without apparent drawbacks. For a visual representation of what I propose to call “Lane-Petter’s Pipeline” (LPP), see Fig. 1.

Lane-Petter held that a steady increase of animal use in biomedical research was practically inevitable for two reasons: First, he viewed model selection as a positive feedback loop: “[T]he more a given species is used in research, the more information about it is collected; and the more is known about a species the more […] it is likely to be used” (Lane-Petter, 1961, p. 3). This seems plausible to an extent, as the attractiveness of a model depends in part on the history of previous use, the availability of infrastructure, and the cost and difficulty of using it (Leonelli & Ankeny, 2012; Dietrich et al., 2020; Green, 2024, pp. 10–12).

Second, Lane-Petter thought that researchers explore new biomedical issues by using animal models first and apply non-animal-models only later in the process, when more specific questions can be asked. For science, Lane-Petter argued, animals’ complexity is both a blessing and a curse:

“The laboratory animal is a complicated and expensive biological system with a large number of variables, many of them controlled with difficulty and some almost beyond control. No scientist would choose such a complicated system if a simpler one would serve his purpose; such as a chemical, a physical, a micro-biological or even a mechanical system.” (Lane-Petter, 1961, p. 132)

Thus, Lane-Petter considered animals to be helpful models in scientific exploration, but clumsy and costly as precision tools. His example referred to the history of vitamins research: To find roughly appropriate dosages that would make vitamin preparations helpful to human patients, experiments were carried out on animals who had been denied the respective vitamin. Only based on those early studies, more specific follow-up questions could be asked about microbiological, chemical, and physical phenomena that account for the observed effects, using “simpler” non-animal assays (1961, p. 133).

With the benefit of hindsight, we know that the overall growth of LPP is neither inevitable nor irreversible. Overall animal experimentation in many countries peaked in the 1970s, then declined and stabilized around the early 2000s (Daneshian et al., 2015; Gärtner, 1999; Stephens et al., 2001; Taylor, 2024). However, Lane-Petter may still have been partially right, since animal experimentation did not decrease uniformly across its various applications. The rise of *in vitro* alternatives for applications in toxicological and pharmaceutical screening, as well as in vaccine production and batch testing, played an important role in driving the overall decrease (Stephens et al., 2001, p. 132; Taylor, 2024, p. 504). Since the early 2000s, many countries have not seen significant decreases in overall animal experimentation anymore, and some have even seen increases (Daneshian et al., 2015; Taylor, 2024; Taylor & Alvarez, 2019). In particular, it is animal use for research purposes that has increased in Europe and the US over the last twenty years, while animal use in regulatory testing and routine production has been decreasing (Taylor, 2024, p. 508). Thus, animal use appears to have decreased primarily where data requirements are clearly defined and fairly stable, but not on the more exploratory, methodologically innovative, and open-ended side of science.

In any case, the number of animal uses within LPP depends on both the entry and the exit rate – on how many animal uses are being replaced by alternatives and how many new ways of using animals for research are being developed. Policies to support replacement aim to accelerate the exit side, but what about the entry? The following two sections will advance two key claims: First, there are multiple avenues for developing new animal models, giving rise to practically infinite room for further innovation (Section 4); and scientists’ model innovation behavior plausibly depends on a range of factors, some of which can be influenced by policy (Section 5).

## Avenues for the innovation of new animal models

Philosophers have discussed a variety of different roles played by animals in science (for overviews, see Ankeny & Leonelli, 2020; Green, 2024). Some animals are used in a non-representational, “instrumental” fashion (Germain, 2014; Green, 2024, pp. 41–44), including as detection devices (e.g., zebrafish used to detect endocrine disrupting chemicals in water, Green, 2024, p. 42) and as sources of material (e.g., horseshoe crabs whose blood is used in vaccine development, Green, 2024, p. 43). Oftentimes, however, animals are used as material models, thus as representational devices or surrogate sources of evidence (Green, 2024, p. 7).

A helpful framework to understand animal models is provided by Ankeny and Leonelli (2020, p. 26), who draw on the more general “DEKI” (denotation-exemplification-key-imputation) account of material models by Frigg and Ngyuen (2018). In the terminology of this framework, “models” are the particular animals (e.g., ten particular mice) in a study. These models represent a target system, such as human beings, particular patients, or some taxon to which the animals themselves belong (e.g., vertebrates). The representational relation is achieved through three crucial steps (Ankeny & Leonelli, 2020, pp. 26–30): First, the models need to *exemplify* properties shared by members of their kind, such as certain genetic pathways or developmental traits. Second, the properties of the model need to bear a relevant *connection* to properties of the target system. This connection is established by assumptions termed the “key,” such as the assumption that the properties in question are shared due to phylogenetic homology or the assumption that these properties are not environmentally determined in uncontrolled ways.^5^ Third, the generic properties of the target system have to be *imputed* to the target system, such as particular human patients.

Applying the DEKI account to animal models can help in conceptualizing avenues for further innovation within animal models: Innovation can (1) expand the range of animals used as models, (2) make tractable more properties exemplified by the model, (3) improve the fit between properties of model and target, (4) investigate new target properties, and (5) expand or refine the target. Consider some examples of each kind of innovation:

First, one can expand the range of animals used as models. Some philosophers of science have been concerned about excessive reliance on a limited range of highly standardized models and have pointed out the potential of lesser used species (Bolker, 2012). For example, Antarctic icefish and dune mice may hold promise as models for certain human diseases (Bolker, 2012; Schartl, 2014), non-human primate models of rare human diseases have become more feasible to identify due to advances in genetic sequencing (Vallender et al., 2023), and gerontologists have become increasingly interested in using killifish and bathyergid mole-rats (Holtze et al., 2021).

Second, one can expand the range of properties that can be tracked in animal models. For example, some animals can literally be made transparent, as was done with adult zebrafish (White et al., 2008) and frogs (Nakajima et al., 2023). Another example is the use of fluorescent proteins in transgenic animals that makes tractable some previously intractable properties related to organelle activity in zebrafish (Choe et al., 2021).

Third, one can improve the fit between properties of model and target. This is often the purpose of genetic modification in animal models (Green, 2024, pp. 13–15), in recent years including the use of CRISPR (Shrock & Güell, 2017). For example, mice have often been gene-engineered to more closely mimic the condition of cancer patients (Sharpless & DePinho, 2006). Model-target fit can also be improved through reverse translation (Green, 2024, p. 20), i.e., through developing animal models based on knowledge from human trials (e.g., about successful treatments in humans, in order to better understand their underlying mechanisms). This is particularly attractive in fields where animal-to-human translation has historically been disappointing, such as in addiction research (Venniro et al., 2020).

Fourth, one can represent new target properties in a model. This is possible when the understanding of the target system has itself evolved. For example, increasing recognition of the importance of the gut microbiome for the human immune system made it possible to innovate new animal studies in which individual strains of human gut bacteria were introduced to the gut microbiome of germ-free mice, which helped to reveal that the microbiome affects cytokine production (Gilbert et al., 2018). In fact, the limitations of animal models can sometimes help to arrive at new understandings of target phenomena. For example, it is challenging to model human anxiety or depression in a rat model, which forces animal researchers to break these phenomena down into more manageable parts, such as perhaps a physiologically tractable kind of stress, leading to a new understanding of the target (Green, 2024, p. 19).

Fifth, one can expand or refine the range of target systems for which animal models are used, which often also requires selecting different models. For example, many studies historically relied on male-only samples of animals, resulting in skewed results that inadequately represented women (Beery & Zucker, 2011). Thus, there is room for innovation in animal studies that use adequately mixed samples to represent all humans, or that exclusively use female animals to better represent women. In the extreme, the target can be refined to a single human patient. For example, animal models called patient-derived xenografts (PDX) can be developed by implanting a particular patient’s tumor cells into an animal (Zanella et al., 2022).

The above options each encompass an intractably high number of potential kinds of innovations – for example, all animal species on Earth could potentially be used as models, every human or group could be chosen as the target system, and any genetic property of the model could be modified. As these options cut across each other, room for innovation becomes practically infinite. Of course, not every potential innovation is likely to be made, as most are presumably uninteresting. Identifying interesting innovations is like finding needles in a haystack – but in a practically infinite haystack, there may well be an intractably high number of needles. Furthermore, model innovations can be interesting not just if they help to address preconceived research problems, but also if they can help to inspire new ones. Animal model innovations may thus help to raise and address myriad novel research problems – and initially, no non-animal model will exist to replace them yet. The practice of using animal models and the perceived necessity to use them can thus be reproduced ad infinitum.

Meanwhile, there is similar innovation potential in models that are not sentient animals – one can vary models and targets, identify or make tractable new properties, and improve the connection between properties of model and target. For example, one could improve the connection between organ-on-a-chip models and complex human targets by integrating multiple organ-on-a-chip systems into a complex human-on-a-chip model (Ingber, 2022; Huang et al., 2024).^6^ New materials and ways of processing them can help to expand the range of organ-on-a-chip models available (Alonso-Roman et al., 2024). And organ-on-a-chip systems can be targeted to specific patients by using their cells (Ingber, 2022).

It is not just animal models, but all material models, that have practically infinite room for further innovation. Science would become more compatible with animal protection over time if model innovation were more concentrated in non-animal-models than in new animal models. The crucial question, then, is what determines the focus of model innovation and how policy could help to steer it in a particular direction without jeopardizing science. In the following, last section of this article, I will draw on the notion of repertoires to suggest that model innovation and model use depend on the built environment of scientific work, which policies could strategically aim to transform.

## Scientific repertoires and model innovation

How likely a model is to be innovated and used depends, in part, on how it fits into its environment. Ankeny and Leonelli have coined the term “repertoires” to refer to “the well-aligned assemblages of skills, behaviors, and material, social, and epistemic components that groups may use to practice certain kinds of science, and whose enactment affects the methods and results of research, including how groups practice and manage research and train newcomers” (Ankeny & Leonelli, 2016, p. 20; see also Leonelli & Ankeny, 2015; but cf. Sample, 2017). What kind of science gets done, and how much of it, they suggest, depends not just on what theories are dominant or what hypotheses get confirmed, but also e.g., on how scientists raise funds, how they share their results, and how they organize their communities (Ankeny & Leonelli, 2016, p. 20). Repertoires are “well-aligned,” thus likely to proliferate, if the factors that scientists can control align with the factors they cannot control (Ankeny & Leonelli, 2016, p. 25). To become widely used, a given model – animal or not – thus does not just need to meet science-internal criteria, but it more generally needs to fit into a successful repertoire.

Empirical research, too, has pointed in the direction of the idea that the extra-scientific environment crucially codetermines what questions scientists ask and which models they use. According to the seashore walk model (Jia et al., 2017; Huang et al., 2023), researchers tend to move to new topics when their work on the previous topic is not (or no longer) rewarded in terms of publications and citations, resembling a person collecting shells at the beach who moves on whenever they do not find any more shells. To be sure, there are differences in how scientists navigate this landscape, so that some are more likely to follow large topics (Li et al., 2017) and some are more willing to take the risk of bold innovations in topic selection (Foster et al., 2015). Still, there is evidence that researchers generally learn to pursue topics that are rewarded by the environment comprising such components as the publication landscape, the funding landscape, and the job market (Huang et al., 2023). Some animal researchers have confirmed this impression in qualitative interviews, emphasizing that changing directions often makes it more difficult to obtain funding and that “publish or perish” culture disincentivizes learning new methodologies (McGlacken & Reed, 2024, pp. 22–23, 29).

Qualitative interviews (Kahrass et al., 2024) and thematic content analyses (Veening-Griffioen et al., 2020) have also suggested that environmental factors, such as having a model readily available and having colleagues with expertise in using it, play an important role in model selection. Dietrich et al. (2020) similarly argue that both epistemic and practical criteria are relevant for what makes a good material model.^7^ For example, a good model needs to provide phenomenal access to the properties to be tracked, needs to be adequately responsive to experimental intervention, and needs to be durable enough to display the behavior at issue (Dietrich et al., 2020, pp. 4–5) – but it also needs to be available, affordable, and convenient to use (Dietrich et al., 2020, pp. 5–7). Overall, the authors put forward a list of twenty criteria, encompassing both epistemic and practical respects in which a model can be better or worse (see Table 1).

**Table 1:** Criteria for organismal choice, adapted from Dietrich et al. (2020, p. 2)

Cluster	Criteria	Plausibly policy-influenced
(A) Access	(1) Ease of Supply	x
	(2) Phenomenal Access	
	(3) Ethical Considerations	
(B) Tractability	(4) Standardization	x
	(5) Viability and Durability	
	(6) Responsiveness	
	(7) Availability of Methods and Techniques	x
	(8) Researcher Risks	
(C) Resourcing	(9) Previous Use	
	(10) Epistemic Resources	x
	(11) Training Requirements	x
	(12) Informational Resources	x
(D) Economies	(13) Institutional Support	x
	(14) Financial Considerations	x
	(15) Community Support	x
	(16) Affective and Cultural Attributes	x
(E) Promise	(17) Commercial and Other Applications	x
	(18) Comparative Potential	
	(19) Translational Potential	
	(20) Novelty	

Many of these criteria describe relations a material model bears to the built environment of scientific work. For example, how easy a model is to supply (criterion 1) depends on how well it fits into existing institutions and infrastructures (e.g., are there institutionalized ways to obtain the model and is there personnel to assist in the process, as in a university’s animal facilities?). To what extent it is standardized (criterion 4) depends on how much previous work has been invested into establishing common vocabulary, protocols, and reporting standards. To what extent methods and techniques are available to use the model (criterion 7) is a matter of infrastructure, devices, and other previous investments. Overall, it appears that roughly half of the criteria on the list (1, 4, 7, 10, 11, 12, 13, 14, 15, 16, 17) are at least partially determined by priorities set by institutions, funders, the scientific community, or other stakeholders. To an extent, human decisions make some models better than others and thus plausibly encourage further innovation in some kinds of models more than in others.

The dependency of model quality on the built environment of research work may provide opportunities for policy interventions. Policy could aim at providing the friendliest environment possible for non-animal-models (e.g., cell cultures, organoids, organs-on-a-chip, computer simulations, human participant studies) with the aim of giving them an edge over animal models and making it more likely that the new best models researchers can innovate are not animals. Thinking in terms of repertoires, the goal would be to transform the conditions of scientific work so that new well-aligned repertoires beyond animal experimentation can emerge.^8^

Transition science provides frameworks to think about potential policy interventions. Understanding the existing environment of scientific work as a “socio-technical regime” (Geels, 2002) comprised of different agents (e.g., scientists, research institutions, funders, journals, regulators, the general public) who stand in more-or-less stable relations, we can think of interventions as attempts to help niche innovations accumulate into stronger trends that can disrupt and reorder the incumbent regime. This approach was used by NCad (2016, Appendices 1–2) to conceptualize a potential transition beyond animal research. Kanger et al., (2020; see also Müller, 2024b) distinguish between six interventions points that can help such transitions along: (1) Stimulate innovation in niches; (2) Accelerate niche innovations into trends; (3) Loosen or revise incumbent regime structures to facilitate change; (4) Address repercussions of regime change; (5) Coordinate between different relevant regimes (e.g., across countries or sectors of the economy) to facilitate change; (6) Tilt the regime’s background conditions (“landscape”) to facilitate change. In Table 2, consider some suggestions for potential policy measures along these intervention points and how they might make non-animal-models better models.

**Table 2:** Potential policy intervention points with examples

Intervention point	Examples	Related criteria for model quality (see Table 1)
(1) Stimulate niches	Earmark resources for innovation of non-animal-models for highly animal-reliant fields	1, 7
	Offer top-up funding for animal-free research projects to encourage innovative use of non-animal-models (see ZonMw, 2024)	14
(2) Accelerate trends	Provide resources for standardization of promising non-animal-models (see e.g., CEN/CENELEC FGOoC, 2024)	4
	Support non-animal-model start-ups through the “valley of death” (NCad, 2016, p. 21)	17
	Invest in infrastructure for promising non-animal-models (e.g., organoid facilities) (McGlacken & Reed, 2024, p. 27)	7, 13
	Support the creation and dissemination of training materials about non-animal-models	12
(3) Loosen/revise incumbent structures	Revise career development training programs to include non-animal-models (McGlacken & Reed, 2024, p. 19)	11
	Support (or even require participation in) “Helpathon” workshops to encourage new collaboration networks (Helpathon, 2023; McGlacken & Reed, 2024, p. 25)	13, 15
	Create programs to connect users of animal and non-animal-models so that knowledge and needs can be communicated (McGlacken & Reed, 2024, pp. 32–34)	15
	Invest in longer-term research funding to enable animal researchers to undertake training (McGlacken & Reed, 2024, pp. 23–24)	11
(4) Address repercussions of regime change	Avoid chaos by creating working groups to coordinate transition activities among different stakeholders (NCad, 2016, p. 5)	13
	Avoid skills mismatch by offering training to animal facilities staff	11, 13
(5) Coordinate across regimes	Coordinate internationally to support the development, validation, and use of non-animal-models	13
	Create transparent pathways for regulatory acceptance of new non-animal-models to incentivize research	17
(6) Tilt the landscape	Symbolically endorse an eventual transition to non-animal-models as a policy goal	13, 16
	Give tax breaks for private investments in line with the transition (e.g., facilities to use non-animal-models) (NCad, 2016, p. 26)	1
	Communicate proactively about non-animal-models	16

Which of these and similar measures are promising and sensible depends on context. In fields where non-animal-models are lacking or underdeveloped (e.g., basic neuroscience, behavioral research), the best option may be to focus activities on niche innovation. In fields where non-animal-models are already more widely used (e.g., toxicology), a greater range of measures across the intervention points may be possible. In any case, while one can list these interventions side-by-side, one cannot successfully take them in isolation. A strategic program would need to assess the state of existing repertoires, the promise of emerging post-animal research practices, and the transformative potential of different policy mixes.

Strategic programs of this kind would aim at strengthening repertoires that are not heavily animal-dependent at the expense of ones that are. Repertoires encompass not just central models, but also research agendas – conceptions of the questions that should be asked and the data that is relevant to address them. While a replacement approach merely substitutes models while keeping the research agenda as-is, this approach would aim at *displacing* one research agenda with another. And once the focus of scientific work is on the kinds of questions non-animal models are fit to answer, and on the kinds of data they are fit to provide, further model innovation is less likely to produce an ever-continuing stream of new animal models. Thus, in addition to the replacement approach, which addresses LPP’s exit side, a “displacement” approach could help to address the entry.

## Conclusion

Replacement efforts have a necessary role to play in making science and animal protection more compatible over time. But on their own, they are not sufficient, because they merely react to the status quo of animal research, which is constantly evolving itself. William Lane-Petter articulated the problem, first using the image of a “pipeline” that animal uses constantly exit, but also simultaneously enter. Room for innovation is practically infinite, both in new animal models and in new non-animal-models. If the goal is to decrease harm to animals in science without holding science back, the built environment of scientific work needs to be tilted toward the innovation of new non-animal-models. Thinking in terms of scientific repertoires can help to envisage what potential policies would probably need to look like. Policy can likely help to achieve this by addressing factors such as funding, infrastructure, and community support, that influence the selection of questions and models. Such policies would not demand that non-animal-models need to *replace* some animal-reliant original by addressing the same research problem. Rather, repertoires that heavily rely on non-animal-models would be enabled to increasingly *displace* heavily animal-reliant repertoires, which includes establishing new research agendas. Anyone seeking to reduce goal conflicts between science and animal protection – whether governments, policy-makers, activists, scholars, or citizens – is thus well-advised to look beyond an approach of merely promoting replacement.

## Data Availability

N/A.

## Footnotes

1 Burch made a passing reference to Lane-Petter’s pipeline imagery (2009 p. 274), but did not elaborate further.

2 That model selection often precedes problem selection does not mean that the converse never happens. In some areas of biomedicine, research problems may often be inspired by disease burden more than by specific experimental systems (or a combination of both).

3 By a “non-animal-model,” I mean a model that is not a sentient animal, even if it relies on materials of animal origin (e.g., animal cells, fetal bovine serum). The reason for this terminology, in line with this section’s argument, is that it avoids casting models other than animal models as mere “alternatives,” while not being as vague as “new approach methodologies.”

4 An even wider definition of replacements, e.g., as “any method that does not use a live animal,” is clearly overinclusive. For example, philosophical thought experiments and particle acceleration experiments would unhelpfully count as replacements. This would make the replacement an unhelpful principle to guide action.

5 One might wonder how so-called “negative models” (Green et al., 2018; Green, 2024, p. 2) fit into the DEKI account. Negative models are organisms that are used as models because they *differ* from humans in crucial features – e.g., naked mole-rats are largely immune to cancer (Green et al., 2018, pp. 9–10). Does this mean that naked mole-rat models represent a human target “negatively” in some way? No. In work with negative models, the questions of interest are about taxa the negative models belong to – e.g., about how animals like naked mole-rats achieve near-immunity to cancer. Humans are not the representational target. Negative models are not odd examples for, let alone counterexamples to, the DEKI account.

6 This responds to the well-known limitations of non-animal-models when it comes to representing intact organisms (LaFollette & Shanks, 1993). This is a concern with model-target connection, similar to the concern that results from animal models often do not translate to human targets. But in either case, the limitation can itself be productive because it encourages new conceptualizations of the target and it can be treated as a prompt for further improvements.

7 Dietrich et al. (2020) focus on model organisms, but their advice generalizes to models that are not organisms, such as cell cultures, organoids, organs-on-a-chip, or computer simulations.

8 One might also say, with Sample (2017), that the way scientists justify their work and link it to desirable futures and notions of a good life are also part of repertoires. So rather than merely encouraging the formation of *new* well-aligned repertoires, policy might aim at producing the *first* repertoires that are well-aligned with new societal values – such as trying to avoid goal conflicts between science and animal protection.
